# Neuroinfections (What Do I Do Now?)

**DOI:** 10.3201/eid1909.130975

**Published:** 2013-09

**Authors:** James J. Sejvar

**Affiliations:** Centers for Disease Control and Prevention, Atlanta, Georgia, USA

**Keywords:** neuroinfections, acute disseminated encephalomyelitis, neurologic infectious diseases, etiology, postinfectious neurologic syndromes

This edition in the series of What Do I Do Now? books provides a nicely organized, concise, and informative guide for the clinician on the approach to the clinical features, differential diagnosis, and management of several neurologic infectious diseases, as well as postinfectious neurologic syndromes such as acute disseminated encephalomyelitis. The illnesses and pathogens covered in this book include those that the average clinician is likely to encounter in routine clinical practice, as well as some uncommon conditions rarely encountered outside the arena of clinical neurovirology.

Each section starts with a brief clinical vignette, describing the history, clinical findings, and other relevant medical data of a particular patient. At the end of the short vignette, readers are instructed to ask themselves what they would do in this particular clinical situation. The following section then goes into greater detail about the differential diagnosis, testing, and management and treatment approaches associated with the particular pathogen. In the process, the reader learns not just about the particular pathogen in question, but about other possible etiologies that may produce a similar clinical situation. Each chapter finishes with key, take-home items regarding the illness, as well as select references relevant to the pathogen/syndrome. The reader is provided with insight into the diagnostic thought process that the practicing neurologist goes through in thinking through issues around neurologic infections.

This book would be most useful to medical students, residents, and other general practitioners who wish to explore further approaches to the patient with an apparent neurologic infection because of its practical, clinical-based approach. Although perhaps less useful as a reference text, it may also serve as a useful addition to the clinician’s library in those instances in which one would like a quick, concise synopsis of particular infections or etiologies that are being suspected in a particular patient. In summary, Neuroinfections (What Do I Do Now?) serves as a helpful and informative summary of major etiologies of neurologic infections, in an enjoyable, easy-to-read format.

